# Applying medicinal chemistry strategies to understand odorant discrimination

**DOI:** 10.1038/ncomms11157

**Published:** 2016-04-04

**Authors:** Erwan Poivet, Zita Peterlin, Narmin Tahirova, Lu Xu, Clara Altomare, Anne Paria, Dong-Jing Zou, Stuart Firestein

**Affiliations:** 1 Department of Biological Sciences, Columbia University, New York, New York 10027, USA; 2 Corporate Research and Development, Firmenich Incorporated, Plainsboro, New Jersey 08536, USA

## Abstract

Associating an odorant's chemical structure with its percept is a long-standing challenge. One hindrance may come from the adoption of the organic chemistry scheme of molecular description and classification. Chemists classify molecules according to characteristics that are useful in synthesis or isolation, but which may be of little importance to a biological sensory system. Accordingly, we look to medicinal chemistry, which emphasizes biological function over chemical form, in an attempt to discern which among the many molecular features are most important for odour discrimination. Here we use medicinal chemistry concepts to assemble a panel of molecules to test how heteroaromatic ring substitution of the benzene ring will change the odour percept of acetophenone. This work allows us to describe an extensive rule in odorant detection by mammalian olfactory receptors. Whereas organic chemistry would have predicted the ring size and composition to be key features, our work reveals that the topological polar surface area is the key feature for the discrimination of these odorants.

## Abstract (web summary)

Understanding the basis of odour perception and discrimination is a challenging task, due to the inherent complexity of the olfactory system. Here, the authors use a medicinal chemistry approach to derive biologically relevant rules for odorant classification.

## Graphical abstract

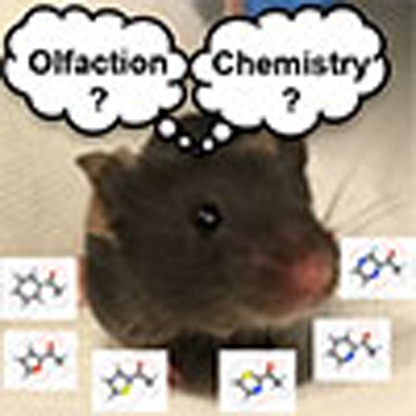

A comprehensive system for classifying odours has been an elusive goal of olfactory inquiry for centuries. The root of the problem, which can be stated most simply as biology versus chemistry, can be seen even in the earliest attempts to bring order to odours. Linnaeus, the master classifier, developed an odour classification scheme using seven primary percepts along a scale of pleasant to unpleasant^1^. Following him, Zwaardemaker^2^ proposed the most comprehensive organization of odours, using 9 or 10 perceptual groupings. With the 19th century development of atomic and organic chemistry, numerous researchers attempted to correlate chemical characteristics with odours^3^,^4^. Perfumers and other fragrance purveyors implemented their own, sometimes less scientific schemes^5^. In the past century more modern attempts generated schemes that encompassed psychophysical descriptors and behavioural responses to complex mixtures^6^,^7^,^8^. However, with the landmark discovery of the unexpectedly large odorant receptor (OR) family of GPCRs by Buck and Axel^9^, these efforts largely came to a halt, replaced by the promise of a molecular basis for odour perception. Because typical mammalian odour gene families number over a thousand different receptors, it seemed that the coding problem would soon be solved with high-throughput screening technologies^10^.

Mature olfactory sensory neurons (OSNs) are believed to express only one OR gene^11^,^12^,^13^. This property, combined with the unexpectedly large number of receptors, has given rise to the widely accepted proposal that peripheral discrimination works through a reciprocal combinatorial code in which one chemical can be detected by different ORs and one OR can detect a group of different chemicals^14^,^15^. Additionally, the axons of all OSNs expressing a particular OR project to the same glomerulus in the olfactory bulb, suggesting a labelled line-style ‘odour-map' in the brain^16^,^17^,^18^. Taken together, these properties seemed to reduce the odour-coding problem to simply matching particular receptors to their cognate odours. Thus, recent efforts have mainly been directed at identifying ligands for various ORs by screening large sets of supposedly diverse odours^19^,^20^,^21^. However, this programme has run into several obstacles.

First, only a handful of ORs have been successfully de-orphaned, severely limiting the possibility of uncovering hypothesized combinatorial rules. Additional confusion arose when an unexpectedly large repertoire of chemically different molecules were identified as ligands of the single mouse OR, SR1 (ref. 22), complicating the idea of an ‘odour-map' and re-opening the question of broadly versus narrowly tuned receptors. Finally, several psychophysical odour paradoxes remain, such as the diversity of compounds that give rise to identical musk percepts. The enormity of the issue was further emphasized by a recent publication claiming that the human olfactory system could discriminate over 1 trillion odours^23^. Absent a systematic understanding of odour detection and discrimination at the periphery, it is difficult to imagine how higher brain centres process the sensory input to develop perceptions and regulate behaviour.

To address these issues from a new perspective, we here take an alternate approach to receptor ligand interactions that is based on medicinal chemistry principles. Medicinal chemistry emphasizes biological function—in this case receptor activation—over chemical form. Similarity between odorants is defined not by strict chemical characteristics but rather by their ability to activate the same receptor or receptors. We use a panel of compounds based on the common odorant acetophenone to investigate the effect of heteraromatic ring substitution for benzene rings on its odour percept. Using both single cell responses and behavioural tests in mice we find that the classification of the odorants is significantly different from the one expected when classified using classical organic chemical rules. From these results it appears that this approach, based on medicinal chemistry and the related concept of bioisosterism, may reveal a novel strategy for comprehending odour discrimination.

## Results

### Responses of OSNs to aromatic odorants

Odorants are multidimensional stimuli but not all features are necessarily equally weighted by ORs. Here, in a calcium imaging assay, we challenged dissociated mouse OSNs with a panel of related heteroaromatic odorants to investigate whether the ring's sterics (size) or its toplogical polar surface area (TPSA) are better correlated with odorant co-detection.

**Figure 1:**
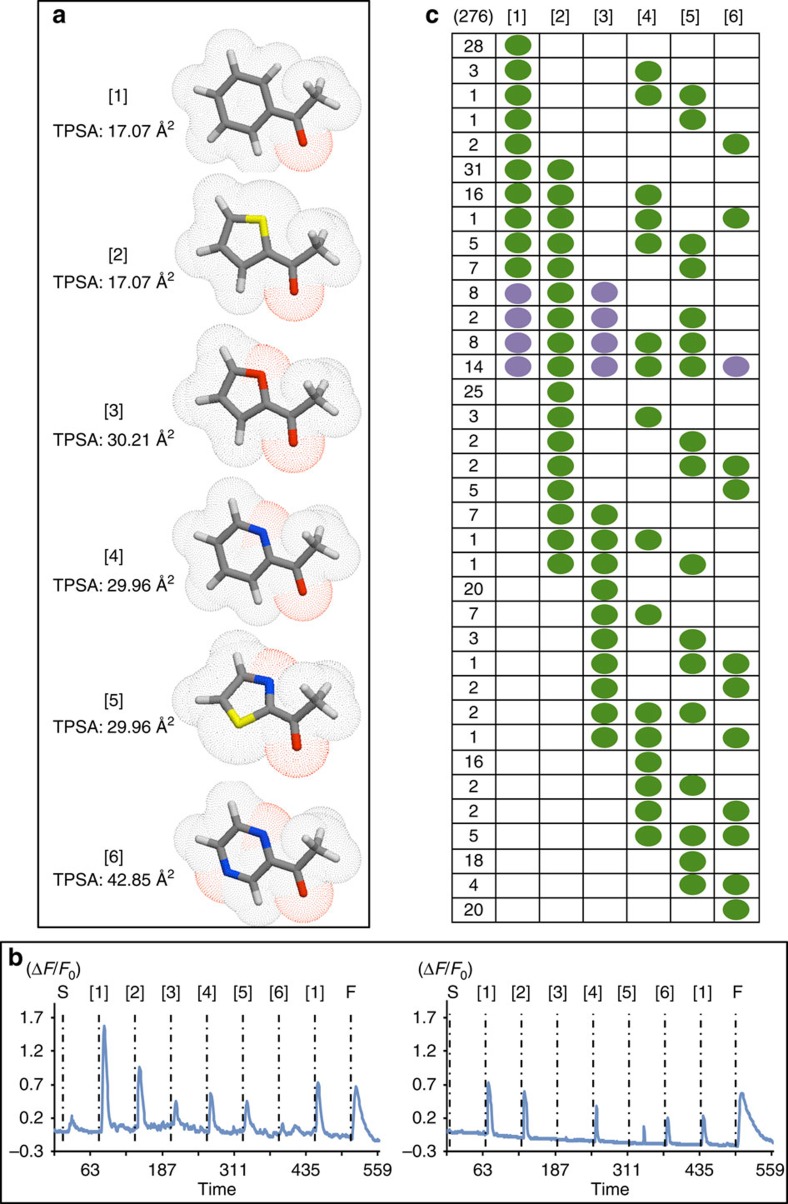
Reponses of dissociated OSNs to Panel 1 odorants in calcium imaging. (**a**) Three-dimensional (3D) representations of Panel 1 odorants. The vertices of the tubes symbolize atoms—grey, carbon; blue, nitrogen; yellow, sulfur; red, oxygen. The dotted red surface around the atoms represents polar regions of the surface area. 3D-representations of these and all odorants used in the study were made using Galaxy 3D Structure Generator free software (www.molinspiration.com.) and TPSA were calculated according to ref. 36. (**b**) Calcium imaging traces of two different OSNs responding to Panel 1 odorants. (**c**) A total of 276 OSNs out of 2,750 viable OSNs responded to at least one Panel 1 odorant, leading to 36 distinct binary response patterns. The numbers indicate how often a particular response pattern was observed. Green dot: activation of the OSN by the corresponding odorant. The OSNs that respond to [1] and [3] always respond to [2] (Purple dot). OSN that respond to [1], [3] and [6] always respond to all the odorants of the panel (Purple dot). S, dimethyl sulfoxide; F, forskolin.

Panel 1 consisted of acetophenone [1] and five derivatives that replaced the apolar, 6-membered benzene ring with heteroaromatic rings of different sizes and atomic composition (Fig. 1a). Ten per cent of viable OSNs (276/2,750) responded to at least one panel member. Thirty-six distinct patterns were observed when responses were conservatively scored in a binary fashion (Fig. 1c). The analogues varied in their ability to mimic [1] in terms of activation. Of the OSNs detecting [1], 72% also detected 2-acetylthiophene [2], 38% detected 2-acetylpyridine [4], 30% 2-acetylthiazole [5], 25% 2-acetylfuran [3] and 13% 2-acetylpyrazine [6]. [1] and [2] have similar TPSAs but different ring sizes. In contrast, [1], [4] and [6] have the same ring size but different TPSAs. That [1] and [2] are far more frequently co-detected than are [1] and [4] or [6] suggests that TPSA is a more heavily weighted ‘epitope' than ring size.

The prioritization of TPSA over ring size appears to be a general trend shaping OSN response patterns. [1], [4] and [6] preserve the same ring size, but as the TPSA increased, the extent of co-detection with [1] decreased. Among OSNs responding to [2], 65% co-detect the similar TPSA but larger-sized ring [1], while only 32% co-detect the higher TPSA but similar-sized ring [5]. Likewise, among OSNs responding to [4], 50% co-detect the similar TPSA but smaller-size ring [5], while only 37% co-detect the higher TPSA but similar-sized ring [6]. This further reinforces that although the geometry of the molecule may be most salient, the TPSA seems to be the driver of these co-recognition patterns.

Strikingly, we found that the diversity of response patterns was constrained by two extensible rules. The first rule is that, at the assay concentration of 30 μM, every OSN that detects both [1] and [3] will also detect [2]. Even when assayed at a higher (150 μM) concentration (Supplementary Fig. 1), this same ‘if [1] and [3] then [2]' rule applies, suggesting that there is a conserved biological constraint among OR-binding pockets. At 30 μM, we also note that [5], a five-membered ringed odorant with similar TPSA to [3], can substitute for [3] 95% of the time in this rule, making the relationship ‘if [1] and [5] then [2]' a highly predictive one. The second extensible rule is that if an OSN detects [1], [3] and [6] then it will detect all the odorants of Panel 1. Although at first surprising, this rule may be considered to be a fusion of the rule ‘If [1] and [3] then [2]' with how OSNs respond to a graded increase in TPSA within a fixed ring size among [1], [4] and [6].

Although the TPSA-based rule was strict for [1], [2] and [3], discrimination based on TPSA partly breaks down when considering [1], [4] and [6]. One might expect that an OSN responding to both [1] and [6] would never reject the intermediate TPSA [4], and yet this occurs 12% of the time. One possibility may be how the appended ketone group interacts with the dual nitrogens of [6]. The benzene ring in [1] has neither a dipole nor a polar constituent. The pyrazine ring in [6] has no dipole (that of the two oppositely situated nitrogens cancelling out), but it is still highly polar (hence its high TPSA). The ketone group, while preferring to lie in plane with the aromatic ring, has freedom to rotate in [1], but less so in [6], where the two polar nitrogens tend to mutually repulse it. With just one nitrogen to interact with the appended ketone, [4] should then co-activate 50% of ORs detecting [1] and 50% of ORs detecting [6]. This is indeed the response pattern observed in the OSNs: 50% of ORs detecting [1] co-detect [4], and 41% of ORs detecting [4] co-detect [6].

To investigate if the rule of ‘If [1] and [3] then [2]' transferred to other contexts, we tested two manipulations. A second panel of odorants (Panel 2) included molecules that were also ketones, but had an extra benzene ring fused to their far end. This manipulation increases the total surface area and affords an extended aromatic system while preserving the TPSA and relationship of the heteroatom to the carbonyl group. Thus, the Panel 2 odorants included 2-acetonaphtone [7] as an analogue to [1], 2-acetylbenzothiophene [8] to [2] and 2-benzofuranyl-methyl-ketone [9] to [3] (Supplementary Fig. 2). Another panel of odorants (Panel 3) replaced the ketone group with a carboxylic acid group. This change allows us to sample a markedly different chemical space as judged by the low frequency of co-recognition of the ketone [1] versus its acid version 2-naphthoic acid [10] (Fig. 1d). Panel 3 includes three acids and their ketone analogues: [10] as the acid analogue to [7], benzo[b]thiophene-2-carboxilic acid [11] to [8] and benzo[b]furan-2-carboxilic acid [12] to [9] (Supplementary Fig. 3).

**Figure 2:**
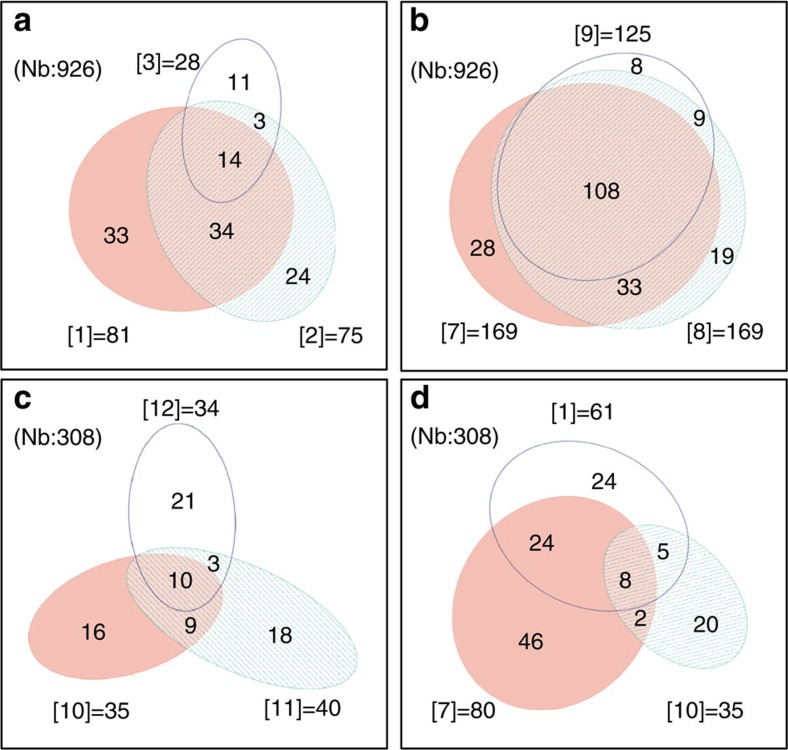
Transferability of the intra-ring TPSA rule. (**a**) Venn diagram of OSNs responding to the single-ring ketones [1], [2] and [3]. If an OSN responds to [1] and [3] it always responds to [2]. (**b**) Venn diagram of OSNs responding to the double-ring ketones [7], [8] and [9]. If an OSN responds to [7] and [9], it always responds to [8]. (**c**) Venn diagram of OSNs responding to the double-ring acids [10], [11] and [12]. If an OSN responds to [10] and [12] it always responds to [11]. (**d**) Venn diagram of OSNs responding to the single-ringed ketone [1], the double-ringed ketone [7] and the double-ringed acid [10]. Note that OSNs co-detecting the acid make up just a small portion of the overall responses. OSNs were counted and converted into surface area for each response combination using the eulerAPE free software. The number of OSNs responding with that pattern is indicated in that sector. All odorants were tested at 30 μM. Nb, total number of viable OSNs screened.

Twenty-six per cent of OSNs (245/926) responded to at least one Panel 2 member, generating 26 distinct binary OSN response patterns. Consistent with the prior study, OSNs detecting [1] co-detected [2] more frequently than [3] (59% versus 17%, respectively). The benzene-fused analogues showed the same trend; OSNs detecting [7] co-detected [8] more frequently than [9] (83% versus 64%, respectively). The strict co-detection rule that was seen for the single ring [1], [2] and [3] also extended to the benzene-fused [7], [8] and [9]. That is, if an OSN responded to both [7] and [9] it always responded to [8] (Fig. 2; Supplementary Fig. 2). Thus, the ‘TPSA rule' is robust among both ketone scaffolds.

Intriguingly, the benzene-fused analogues of Panel 2 activated markedly more OSNs than did their single ring counterparts. Eighteen per cent of OSNs were activated by [7] versus 9% by [1], 18% [8] versus 8% [2] and 13% [9] versus 3% [3] (Supplementary Fig. 2). This suggests that increased surface area and/or extended aromaticity could be a stabilizing factor, perhaps by improving pi–pi stacking with aromatic amino-acid side chains in the binding pocket. This may form the basis of a strategy to rationally design an aromatic odorant to increase the breadth of ORs it targets.

Among the acids of Panel 3, we again observed conservation of the ‘TPSA rule' with all OSNs that responded both to [10] and [12] also responding to [11] (Fig. 2c). Twenty-two distinct patterns were observed when responses were conservatively scored in a binary fashion (Supplementary Fig. 3). Of the 308 OSNs recorded, 45% responded to at least one Panel 3 member; for the ketones 26% responded to [7] and 20% to [1]. For the acids only 11% responded to [10], 13% to [11] and 11% to [12]. These results indicate that acids are generally weaker odorants than ketones. Interestingly the OSNs recognizing the acid [10] were mostly distinct from the population responding to either the single-ringed ketone [1] or the double-ringed ketone [7] (Fig. 2d), lending further support to the transferability of the ‘TPSA rule'.

### Comparing odorant classifications

Medicinal chemistry substitutions can be discrepant in form but they nevertheless preserve similar biological functionality across multiple targets. In our panels, several of the heteroaromatic substitutions from the ‘lead' odorant [1] were inspired by medicinal chemistry substitutions. We thus compared the classification of the Panel 1 odorants using both traditional chemistry-centric and biology-centric approaches.

**Figure 3:**
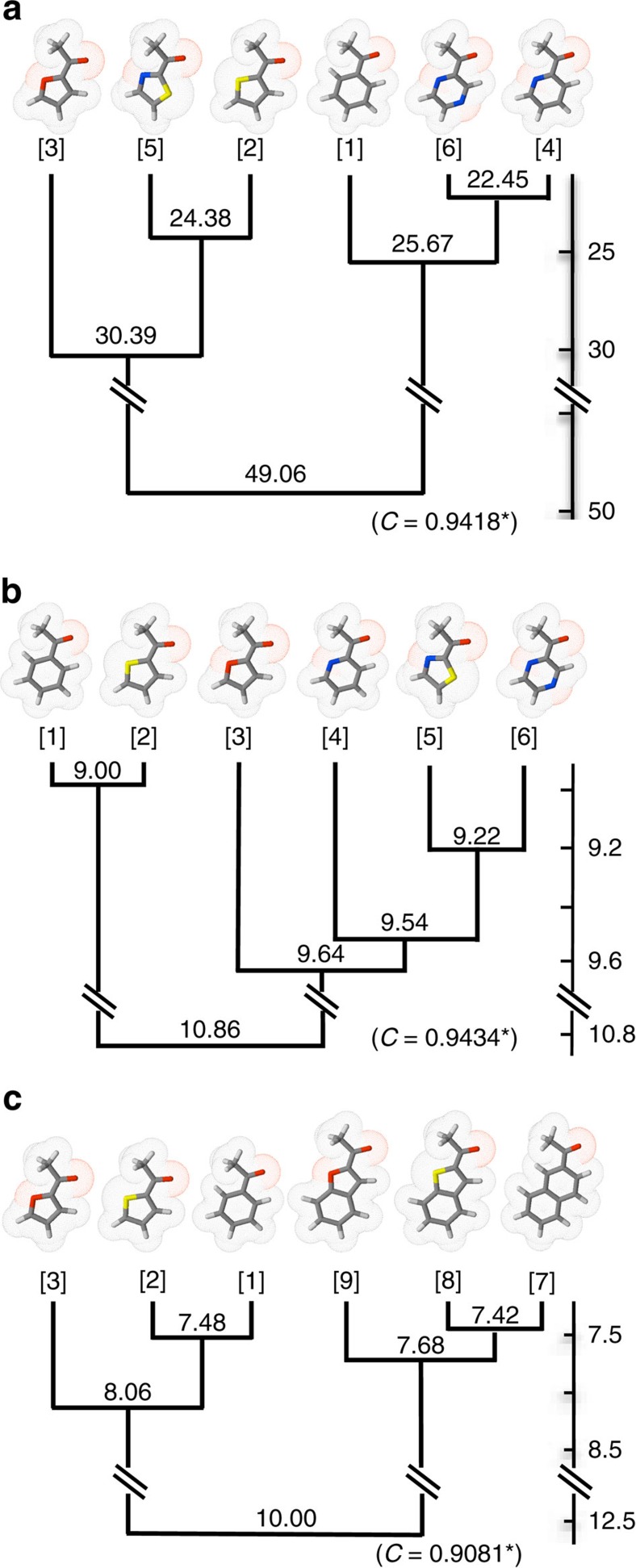
Hierarchical clustering analysis of Panel 1 and Panel 2 odorants. (**a**) Panel 1 odorants clustered according to chemical similarity as evaluated by 1,666 molecular descriptors downloaded through the e-dragon applet. (**b**) Panel 1 odorants clustered according to biological response similarity based on calcium imaging of dissociated OSNs. Note that in the chemical-based clustering the major division is on ring size while in the biological-based clustering the major division is on the TPSA. (**c**) Panel 2 odorants clustered according to their biological responses, as in **b**. Although there is a major division based on the presence of a double-ring scaffold, within each branch further subdivisions follow the TPSA rule as in **b**. *Cophenetic correlation coefficient. All distances in the dendrograms are Euclidian. See online methods for details of dendrogram generation.

For the chemistry-centric approach, we used the e-Dragon software to obtain 1,666 molecular descriptors for each odorant. We generated a dendrogram (Fig. 3a) which revealed two clearly distinguishable branches. The segregation was driven by ring size with the 6-membered ring [1], [4] and [6] forming one cluster, and the 5-membered ring [2], [3] and [5] forming a second cluster. The two families were further fractionated by atomic composition via the presence of nitrogen in the 6-membered ring family and sulfur in the 5-membered ring family. In the 5-membered ring family, [3] and [5] are split apart despite their similar TPSA, leaving [5] to cluster with [2]. This clustering pattern underscores that in the traditional chemistry-centric classification atomic composition is given pre-eminence over TPSA.

For the biology-centric classification the response patterns of the OSNs formed the basis for the hierarchical cluster analysis. The resulting dendrogram has striking differences (Fig. 3b). Notably, in the dendrogram for Panel 1, [1] and [2] are tightly linked, as determined from their biological activity profiles. This branch, which contains the two low TPSA rings, segregates from the odorants with larger TPSA values. TPSA, however, is not the sole determinant of the remaining organization as [5] clusters with the higher TPSA [6] instead of the matched TPSA [4]. When Panel 2 odorants were clustered via their OSN response patterns, the major split was along the lines of total surface area with all the double-ringed odorants segregating from the single-ringed odorants (Fig. 3c). Within each family, however, clustering reflected the division of low TPSA from high TPSA that was seen in the Panel 1 odorants. That is, [1] was tightly linked to [2] and separate from [3], whereas [7] was tightly linked with [8] and separate from [9].

Similarly, biology-centric classification separates Panel 3 odorants according to their TPSA. The three acids segregate from the ketone [7], and [10] was tightly linked to [11] and separated from [12] (Supplementary Fig. 4). A chemistry-centered approach on the other hand separates once again Panel 3 odorants according to their ring size and composition: [7] and [10] group together despite their functional group difference, and separate from [11] and [12].

### Behavioural response of mice to the odorants

Having examined how OSNs parse heteroaromatic odorants, we turned to a habituation–dishabituation test to investigate how readily a mouse could discriminate between select pairings of Panel 1 and Panel 2 odorants. Habituation is defined by a progressive decrease in olfactory investigation towards repeated presentation of the same odour stimulus. Dishabituation is defined by reinstatement of olfactory investigation when a novel odour is presented.

**Figure 4:**
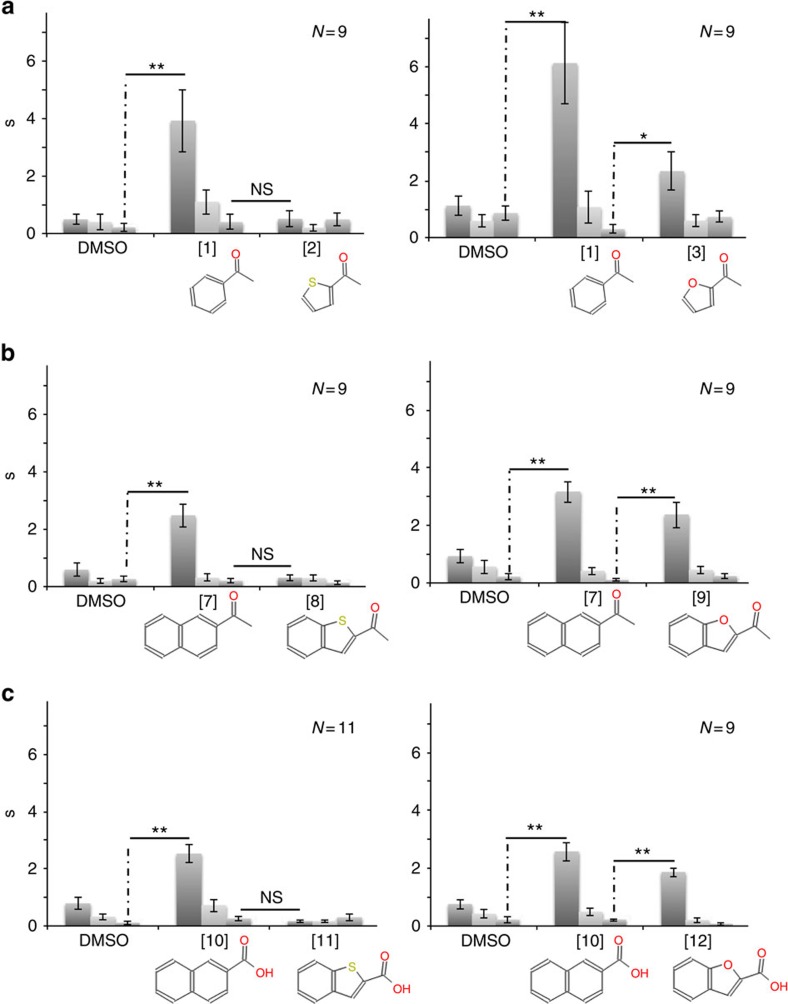
Habituation–dishabituation olfactory test. The average olfactory investigation time (s) by mice during repetitive 2 min exposures to odorant pairs or DMSO (solvent). (**a**) Mice that habituated to [1] remained habituated to [2] but dishabituated to [3]. (**b**) Mice that habituated to [7] remained habituated to [8] but dishabituated to [9]. (**c**) Mice that habituated to [10] remained habituated to [11] but dishabituated to [12]. In all cases, the analogue with the low TPSA thiophene ring was not discriminated from the lead, low TPSA benzene-ringed version, but the analogue with the higher TPSA furan ring was. Behaviour tests were analysed using ANOVA test followed by a *post hoc* paired *t*-test (*P<0.05, **P<0.005 paired *post hoc t*-test). NS, not significant, *P-value<0.05, **P-value<0.005 paired *t*-test. Error bars: s.e.m. *N*, number of animals.

Many of the trends seen in the behavioural assay paralleled those seen in the response patterns of OSNs. Notably, there was robust acceptance for a carbon-to-sulfur swap; mice that habituated to [1] remained habituated to [2] (Fig. 4). This habituation also occurred when odorants were presented in the reverse order (i.e., habituation to [2] then probed with [1]). Reciprocal habituation also occurred when mice were challenged with [7] and [8], the double-ringed analogues of [1] and [2].

Mice also demonstrated reciprocal habituation to [4] and [5] (Table 1). Like [1] and [2], [4] and [5] are related by a carbon-to-sulfur swap but in an overall more polar background. Although [1] and [2] cluster tightly in the OSN response-based dendrogram, [4] and [5] are admittedly more distant (Fig. 3b). Still, they are far closer in the OSN-based response dendrogram than in the molecular descriptor-based dendrogram. This supports that OSN response patterns are indeed a better predictor of olfactory-guided behaviour.

Clear reciprocal dishabituation was noted for certain carbon-to-oxygen and carbon-to-nitrogen swaps. Mice stimulated by [1] failed to generalize to the oxygen-containing [3]. This behaviour may find its basis in that OSNs show a far lower degree of co-detection between [3] and [1] as opposed to [2] and [1]. Dishabituation was also seen when the mouse was stimulated by [7] but probed with [9], the double-ring analogues of [1] and [3], or stimulated by [10] but probed with [12] (their acids analogues) (Fig. 4). For carbon-to-nitrogen swaps, reciprocal dishabituation was observed between [2] followed by [5] and by [1] followed by [4] (Table 1). Within both of these pairings, the ring size is preserved but TPSA changes. This further reinforces the relative pre-eminence of TPSA from a biological standpoint.

Interestingly, not all the habituations were reciprocal. Habituation was observed when the mouse was stimulated with [4] then probed with [3] but not if stimulated with [3] then probed with [4]. The same situation occurred between [5] and [6]. Cases of asymmetrical habituation have been previously reported in the psychophysical literature and are suggestive of non-overlapping sets of receptors that bind the same ligands.

## Discussion

The classification of the vast number and diversity of odorant molecules has been a controversial topic in psychophysics and more recently in molecular physiology and systems biology of the olfactory system^23^,^24^. Here, our work reveals an extensible rule of odorant detection by OSNs.

In colour perception there is a generally agreed-upon set of rules determining how wavelengths mix to produce millions of hues. In the auditory system the combination of frequencies and amplitudes produces a predictable perception of tonality. No such agreement or scheme is available in olfaction and it remains virtually impossible to predict, from looking at a chemical structure, whether a molecule will have an odour or not, let alone what that quality may be.

One obstacle to gaining this understanding may be that we have adopted a physical and organic chemistry scheme of molecular description and classification. Chemists classify molecules according to characteristics that are useful in synthesis or isolation, features that may be of no importance to a biological sensory system, either at the olfactory receptor level or at higher perceptual levels.

It has been shown that among all molecular features that describe a compound, some are more important than others for odorant perception by receptors^25^,^26^. Learning how features of odorants are weighted by ORs could clarify the fundamental structure of the stimulus space and help predict similarity of odour quality. Computational models such as the 3D-QSAR can already efficiently identify a few key descriptors common to all the ligands of an OR and then predict and design new ligands for that OR^26^,^27^. But this model depends on already partially deorphanized ORs, and these key descriptors appear to be different for every OR. Recently Sobel's lab^28^, correctly identified the problem of odour perception as one of quantifying odour characteristics, has taken a mathematical approach to reduce complex odour structures to a small number of vectors. Unfortunately, a reliance on chemical descriptors means elements of the vectors cannot often be identified with any empirical odour structure. (Examples from their Table 1 include, ‘the molecular multiple path count number', the ‘spectral moment from edge adj. matrix weighted by dipole moments' and 19 other similarly esoteric descriptors).

We have instead taken an approach that is more bio-centric using principles developed in the practice of medicinal chemistry to identify biologically relevant features of an odour stimulus^29^. This is sometimes known as bioisosterism—the practice of exchanging molecular fragments that subtly tweak but largely preserve chemical structure and performance at a variety of enzyme and receptor targets.

As a proof of principle we assessed one type of bioisosteric exchange, that of heteroaromatic rings for benzene, against the suite of mouse ORs. Starting from acetophenone as the ‘lead' odorant, we found that several of the predicted exchanges were, indeed, well tolerated. Acetophenone possesses a benzene ring that can be replaced by alternative ring structures. The most common prediction would be that odour quality varies according to ring steric size and shape or atomic composition. On the contrary, our analysis revealed that the overall TPSA was of greater importance, such that having a ring component with a high TPSA was a generally disfavoured epitope for OSNs responding to acetophenone. These findings at the sensory neuron/receptor level transferred to behavioural testing

A second panel, which included benzene-fused double-ringed versions of the analogues, surprisingly revealed that the double-ringed odorants activated far more OSNs than did the single-ringed ones. The added benzene ring not only increased the breadth of activation across the suite of ORs but it also often led to an increased breadth of tuning for a given single OR. This strategy could be exploited to probe binding pocket accommodation.

A third panel used acid analogues of the double-ringed ketones of Panel 2. We observed only minor levels of co-recognition between the single-ring acid [10] and the analogous single-ringed ketone [1] or the double-ring ketone [7], demonstrating that the acids of Panel 3 likely cover distinct sectors of chemical space than do the ketones of Panel 1 and Panel 2. Yet despite this, the ‘TPSA rule' translated well, demonstrating its robustness as a predictive tool.

An important caveat to this work is that we used a simple binary accounting for whether an OSN was activated or not, and each panel was conducted at a single concentration (with the exception of a control experiment run at 150 μM, Supplementary Fig. 1). Thus, we did not measure affinity or efficacy as variables. Increasing concentration would likely activate additional receptors and alter the patterns of overlap, although it has been shown that increasing concentration only rarely alters odorant perceptual quality^30^. Although these effects are not uninteresting they would have clouded the main purpose of the present study—to determine the biologically most relevant attribute of related molecules among a group of receptors. In this regard the olfactory system offers a novel forum for evaluating medicinal chemistry strategies because we are not testing various molecules on a single receptor, as is the case in pharmaceutical experiments. In the olfactory system we have a large number (>1,000 in mouse) of G-protein-coupled receptors that are being tested simultaneously with a panel of carefully altered odour compounds. In a sense we are using the receptors simply to ‘take a vote,' which is necessarily binary, on the biologically relevant characteristics of a molecule.

Although we can draw no conclusion as to why TPSA should be of special importance there are several interesting speculations. The TPSA is effectively a measure of the solvent accessible surface area presented by a molecule. Given that odorants must pass through both aqueous and lipid environments to access the presumptive binding regions of the receptors, the surface area could raise or lower the entropic cost of accessing that activating region^31^. Access to the receptor, or specific parts of it, may be more crucial in determining the efficacy of a molecule than the particular fit it may make in a presumptive binding pocket. The popular lock and key model of receptor ligand interactions is too naive to capture the biophysical requirements that play a role in how a molecule may interact with and stabilize an activated conformation of the receptor. Bioisosterism is an empiric method for probing and understanding those functional details.

As a bonus, this approach also revealed an extensible rule—that if an OSN accepted both the low TPSA, 6-membered benzene ring and the high TPSA, 5-membered furan ring, then it will always accept the ‘intermediate challenge' of a low TPSA, 5-membered thiophene ring. We witnessed this for ketone odorant sets [1], [3], [2] and [7], [9], [8] and acids [10], [11], [12]. This rule joins the electronegativity rule of ‘if an OSN accepts a n-alcohol and the homologous n-acid, it will always accept the electronegative intermediate homologous n-aldehyde'^32^, and the backbone continuity rule of ‘if an OSN accepts a chain length of N and N+2 in an n-odorant, then it will always accept a chain length of N+1' (refs 15, 33, 34). These three rules show that constraints in detection exist, despite the wide diversity of odorants and receptors.

We anticipate that there are other rules to be discovered through application of this medicinal chemistry strategy, and that these rules may be extended to other, non-olfactory, GPCRs. Notions such as broad versus narrow tuning of receptors could be revisited in terms of sensitivity to molecular features rather than molecular compounds. Indeed it might well be worth revisiting the idea of odour primaries (as in colours or fundamentals in sound) that are recombined in innumerable, but comprehensible, ways to provide a rich odour world.

## Methods

### Chemicals

Two panels of six ketone odorants (Panel 1 and Panel 2) and a panel of three acid odorants (Panel 3) were designed to test the hypothesis that, among odorants, heteroaromatic rings can substitute for benzene rings with ORs exhibiting a predictable preference between them. All panels are derived around a lead odorant, acetophenone [1]. Panel 1 consisted of acetophenone [1], 2-acetylthiophene [2], 2-acetylfuran [3], 2-acetylpyridine [4], 2-acetylthiazole [5] and acetylpyrazine [6]. Panel 2 consisted of acetophenone [1], 2-acetylthiophene [2], 2-acetylfuran [3], 2-acetonaphthone [7], 2-acetyl-benzothiofene [8] and 2-benzofuranyl-methyl-ketone [9]. Panel 3 consisted of acetophenone [1], 2-acetonaphthone [7], 2-naphthoic acid [11], benzo[b]thiophene-2-carboxylic acid [11] and benzo[b]furan-2-carboxylic acid [12]. Odorants [1]–[9] were purchased from Sigma-Aldrich (St Louis, MO, USA). Odorants [10]–[12] were purchased from Acrōs Organics (Thermo Fisher Scientific, New Jersey, USA). Odorant stocks were made in >99% dimethyl sulfoxide (DMSO) (Sigma-Aldrich) and were diluted in freshly prepared Ringer's solution to a final concentration of 30 or 150 μM just before experiments.

### Animals and tissue collection

All animal procedures conformed to Columbia University guidelines for care and use of animals. *OMP-Cre*-driven *GCaMP3* mice used in this work were generated by crossing the *OMP-Cre* line (*JAX 006668*) with the *Ai38* line *(RCL-GCaMP3, JAX014538*). In these compound mutant mice, the expression of the genetically encoded calcium sensor GCaMP3 is restricted to the mature olfactory sensory neurons. All mice were reared and maintained in the department animal facility.

Olfactory sensory neurons were isolated from 5 to 8-week old *OMP-Cre*-driven *GCaMP3* male mice with a genotype of *OMP-Cre*^*+/−*^*GCaMP3*^*−/−*^. The mice were overdosed with anaesthetics (ketamine 90 mg kg^−1^; xylazine 10 mg kg^−1^, i.p.) and decapitated. The head was cut open sagitally and the septum was removed to expose the medial surface of the olfactory epithelium and turbinates. The olfactory epithelium and turbinates were dissected and collected in divalent-free Ringer's solution (mM: 145 NaCl, 5.6 KCl, 10 Hepes, 10 Glucose, 4 EGTA, pH 7.4). The tissue was incubated at 37 °C for 45 min in 5 ml of divalent-free Ringer's solution containing 0.5 mg ml^−1^ collagenase, 5 mg ml^−1^ bovine serum albumin (Sigma-Aldrich), 8 U ml^−1^ dispase (Roche, Bassel, Switzerland) and 50 μg ml^−1^ deoxyribonuclease II (Sigma). The tissue was then transferred to a clean tube of culture medium and washed. The OSNs were dissociated by tapping the tube containing the tissue. The OSNs (50 μl volume) were split onto four concanavalin-coated glass coverslips (Sigma-Aldrich, 10 mg ml^−1^), placed in 35 mm Petri dishes. After allowing the cells to settle for 20 min, 2 ml of culture medium was added to each dish and the dishes were placed at 37 °C for at least 1 h. Culture medium consisted of DMEM/F12 (Gibco BRL, Grand Island, NY, USA) supplemented with 10% fetal bovine serum, 1 × insulin-transferrin-selenium (Gibco BRL), 100 U ml^−1^ penicillin and 100 μg ml^−1^ streptomycin (Gibco BRL) and 100 μM ascorbic acid (Sigma-Aldrich).

### Calcium imaging recording

After being washed with fresh Ringer's solution, the coverslips were mounted on a recording chamber. Imaging was carried out at room temperature on an inverted fluorescence microscope (IMT-Olympus, Tokyo, Japan) equipped with a SIT camera (C10600, Hamamatsu Photonics, Hamamatsu, Japan), a Lambda XL light source (Sutter Instrument, Novato, CA, USA), and Lamba-10B optical filter changer (Sutter Instrument). Using a 1260 Infinity HPLC system (Agilent Technologies, Santa Clara, CA, USA) the dissociated OSNs were stimulated with the odorants in random order between two flanking stimulations with the lead odorant, [1]. A final stimulation with a 10 μM Forskolin (Sigma-Aldrich) solution was made to assess the viability of the OSNs. Recordings were made at 490 nm excitation and 520 nm emission. Images were taken every 4 s and there was a 4 min delay between stimulations. The images were then computed using Metamorph Premier software (Molecular Device LLC, Downingtown, PA, USA) and the cells were manually counted.

### Data analysis of calcium imaging recording

1,666 molecular descriptors for the P1 and P2 odorants were downloaded through e-dragon free applet (http://www.vcclab.org/)^35^. Normalized descriptors were used for calculating Euclidean distances and for generating dendrograms using Matlab (MathWorks, Boston, MA, USA). Neuron responses to Panel 1 or Panel 2 odorants in calcium imaging were transformed to an m*n bool matrix where ‘*m*' is the number of neurons responding to at least one chemical, and ‘*n*' is the number of chemicals used; ‘1' means ‘response' and ‘0' means ‘no response'. This matrix was used to calculate Euclidean distances and generate dendrograms of the odorants using Matlab. A Coshran's *Q* test comparison followed by *post hoc* McNemar tests was performed to compare the odorant ‘response', ‘No response' heatmaps using Statview (SAS institute, Cary, NC, USA).

### Habituation–dishabituation behavioural test

Similarities in perceptual odour quality among the Panel 1 odorants were evaluated by a habituation–dishabituation olfactory test in the mouse. Thirty minutes before experimentation, 5–8 weeks old *OMP-Cre*^*+/−*^ *GCaMP3*^*−/−*^ male mice were placed individually into a hood in an empty mouse cage containing a cotton swab soaked in 1/1,000 DMSO/Ringer's solution. Each animal was then stimulated three consecutive times over 2 min with the DMSO/Ringer's solution soaked cotton swab as a negative control. Then they received three consecutive presentations of a cotton swab soaked in the first odorant solutions at 30 μM. Each presentation lasted 2 min with a 1 min interval between presentations. Following a 1 min rest, animals were then given three presentations of the second odour in a similar manner. Following a final 1-min break, a 30 μM solution of propyl-valerate was given in a 2-min single stimulation as a positive control. The cumulative sniffing time of the cotton swab was recorded using a silent clock. An analysis of variance (ANOVA) statistic comparison, followed by *post hoc* Paired *t*-test, was performed on the results using Statview. Each mouse was used only once with the same odorant. Mice that were unable to detect the first odorant stimulation or that responded to the negative control were removed from further analysis.

## Additional information

**How to cite this article:** Poivet, E. *et al*. Applying medicinal chemistry strategies to understand odorant discrimination. *Nat. Commun.* 7:11157 doi: 10.1038/ncomms11157 (2016).

## Supplementary Material

Supplementary Information Supplementary Figures 1-4

## Figures and Tables

**Table 1:** OSNs discrimination and behaviour response to Panel 1 odorants.

**Odorant pairs**	**Changes**	**Co-activation**	**Exclusion (%)**	**Behaviour**
[1] versus [2]	C–>S, polarity and ring size change	72%/66%	30	Habituated
[1] versus [3]	C–>O, polarity, ring size and TPSA changes	25%/41%	69	Dishabituated**
[1] versus [4]	C–>N, polarity and ring TPSA change	38%/55%	71	Dishabituated*
[1] versus [5]	C–>S, C–>N, dipolarity, ring size and TPSA changes	30%/49%	55	—
[1] versus [6]	C–>N (2 × ), ring TPSA changes	13%/29%	74	Dishabituated*
[2] versus [3]	S–>O, polarity and ring TPSA changes	30%/53%	62	—
[2] versus [4]	Polarity, ring size and TPSA changes	35%/55%	57	—
[2] versus [5]	C–>N, dipolarity and ring TPSA change	37%/65%	53	Dishabituated**
[2] versus [6]	Polarity, ring size and TPSA changes	16%/37%	81	—
[3] versus [4]	Polarity, ring size change	43%/38%	60	Dishabituated*
[3] versus [5]	O–>N, C–>S, dipolarity	40%/40%	60	—
[3] versus [6]	Ring size and TPSA changes	23%/30%	73	—
[4] versus [5]	C–>S, dipolarity, ring size change	42%/47%	55	Habituated
[4] versus [6]	C–>N, polarity, ring size and TPSA changes	26%/39%	68	—
[5] versus [6]	Dipolarity, ring size and TPSA changes	33%/44%	62	Habituated

TPSA, topological polar surface area.

This table recapitulates co-activation among the OSNs and behavioural responses observed with Panel 1 odorant pairs. ‘Changes' gives the substitutions and transformations from one odorant to the other. The first co-activation number gives the percentage of OSNs responding to the first odorant of the pair that were co-activated by the second odorant. The second co-activation number gives the percentage of OSNs responding to the second odorant of the pair that were co-activated by the first odorant. ‘Exclusion' numbers give the percentage of OSNs that respond exclusively to one odorant of the pair among the total number of OSNs that respond to those two odorants. ‘Behaviour' recapitulates the results observed during the habituation/dishabituation olfactory tests with the odorants of the pair. Behaviour results were analysed using ANOVA test followed by a *post hoc* paired *t*-test (*P<0.05, **P<0.005 paired *post hoc t*-test).
