# Supplementary material for: Applying medicinal chemistry strategies to understand odorant discrimination
Source: Nat Commun. 2016 Apr 4;7:11157. doi: 10.1038/ncomms11157 (PMC4822015; doi:10.1038/ncomms11157)
Supplement: Supplementary Information — Supplementary Figures 1-4 [file ncomms11157-s1.pdf]

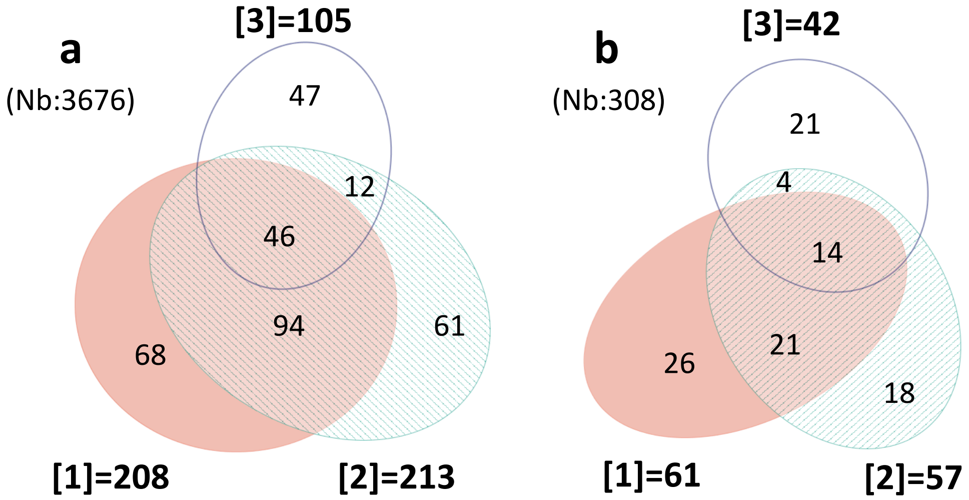

Supplementary Figure 1: Maintainance of the intra-ring PSA rule at high concentrations

(**a**) Venn-Diagram of OSNs responding to 30µM each of the single-ring ketones [1], [2], and [3]. (**b**) Venn-Diagram of OSNs responding to 150µM each of [1], [2], and [3]. Even at the higher concentration, if an OSN responds to [1] and [3] it always responds to [2]. OSNs were counted and converted into surface area for each response combination using the eulerAPE free software. The number of OSNs responding with that pattern is indicated in that sector. [1]: acetophenone, [2]: 2-acetylthiophene, [3]: 2-acetylfuran. Nb: total number of viable OSNs screened.

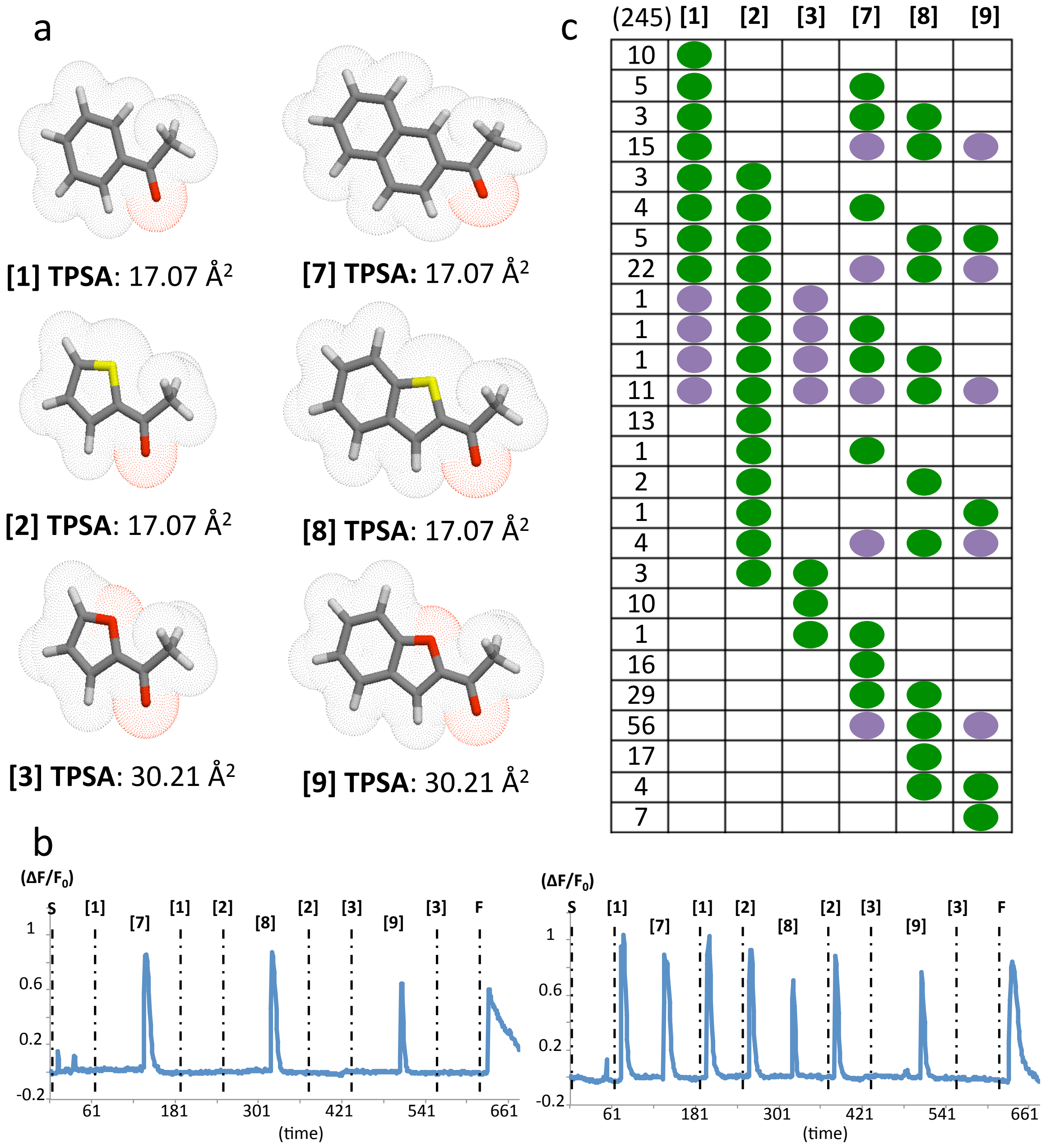

<u>**Supplementary Figure 2: Responses of dissociated OSNs to P2 odorants in Calcium Imaging.**</u>

(**a**) 3D-representation of Panel 2 odorants, following the scheme outlined in Figure 1. (**b**) Calcium imaging traces of three different OSNs responding to Panel 2 odorants. (**c**) 245 OSNs out of 926 viable OSNs responded to at least one

Panel 2 odorant, leading to 26 distinct binary response patterns. The numbers indicate how often a particular response pattern was observed. Green dot: activation of the OSN by the corresponding odorant. The OSNs that respond to [1] and [3] always respond to [2] (Purple dot). The OSNs that respond to [7] and [9] always respond to [8] (Purple dot) S: dimethyl sulfoxide, F: forskolin, [1]: acetophenone, [2]: 2-acetylthiophene, [3]: 2-acetylfuran, [7]: 2-acetonaphthone, [8]: 2-acetyl benzothiophene [9]: 2-benzofuranyl methyl ketone.

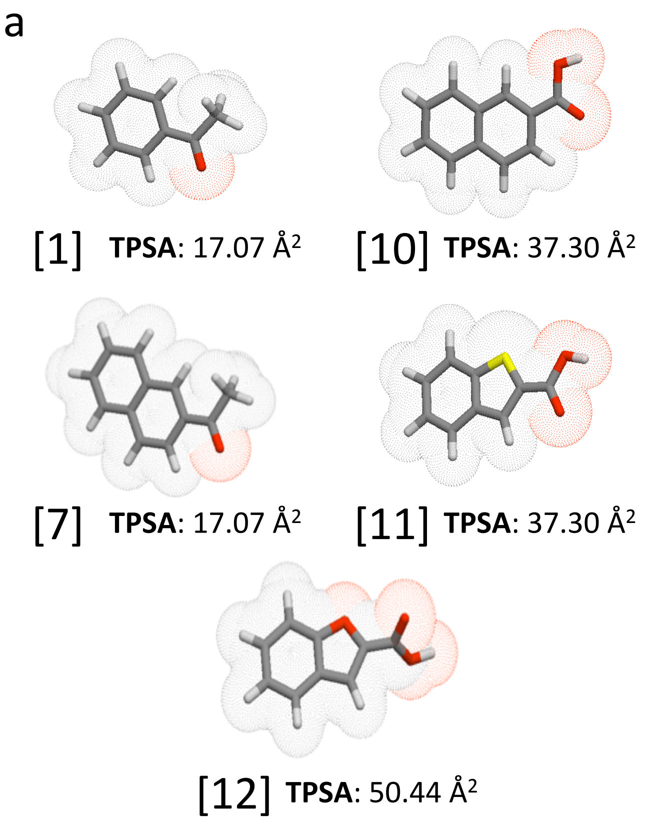

b

(160)	[7]	[10]	[11]	[12]	[1]
40	●				
1	●			●	
23	●				●
4	●			●	
1	●		●	●	
1	●			●	●
1	●	●			
3	●	●			●
1	●	●	●		
1	●	●	●		●
4	●	● (purple)	●	● (purple)	●
12		●			
3		●	●		●
4		●	●		
4		● (purple)	●	● (purple)	
2		● (purple)	●	● (purple)	●
15		●	●		
3			●		●
1			●	●	
1			●	●	●
15				●	
20					●

Supplementary Figure 3: Responses of dissociated OSNs to Panel 3 odorants in Calcium Imaging.

(**a**) 3D-representation of Panel 2 odorants, following the scheme outlined in Figure 1. (**b**) 160 OSNs out of 308 viable OSNs responded to at least one Panel 3 odorant, leading to 22 distinct binary response patterns. The numbers indicate how often a particular response pattern was observed. Green dot: activation of the OSN by the corresponding odorant. The OSNs that respond to [10] and [12] always respond to [11] (Purple dot). [1]: acetophenone, [7]: 2-acetonaphthone, [10]: 2-naphthoïc acid, [11]: benzo[b]thiophene-2-carboxylic acid, [12]: benzo[b]furan-2-carboxylic acid.

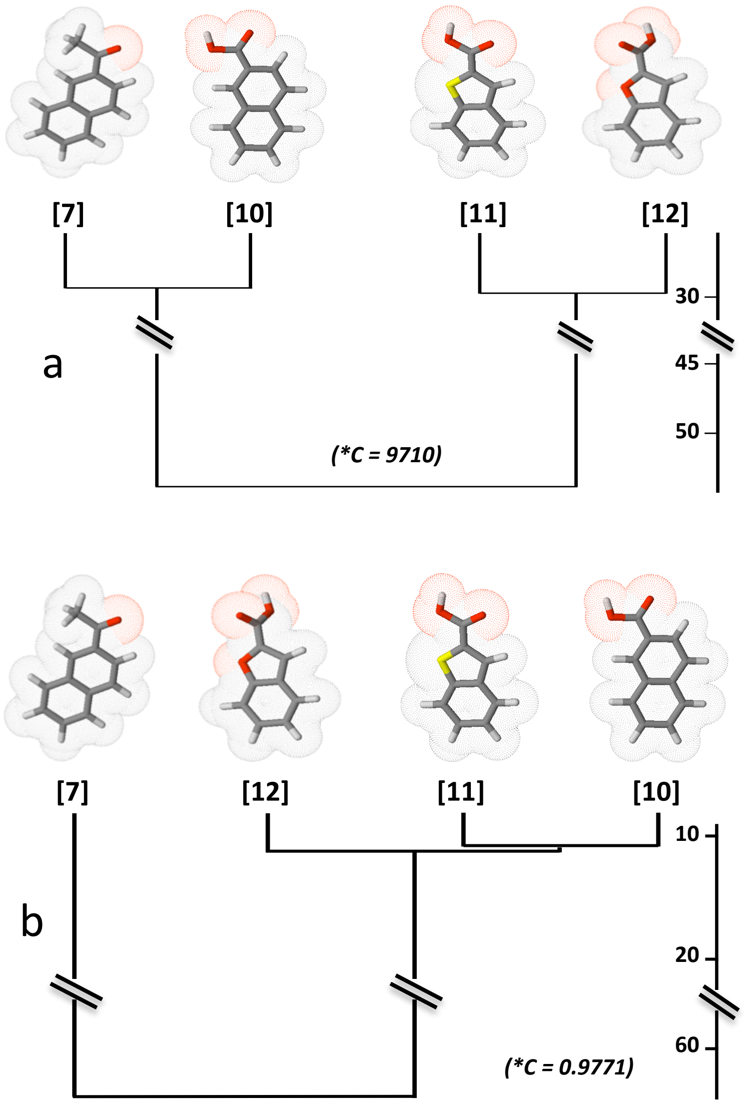

<u>Supplementary Figure 4: Hierarchical Clustering Analysis of Panel 3 odorants</u>

(**a**) Panel 3 odorants clustered according to chemical similarity as evaluated by 1,666 molecular descriptors downloaded through the e-dragon applet. Normalized descriptors were used for calculating Euclidian distances. (**b**) Panel 3 odorants clustered according to biological response similarity as based on calcium imaging of dissociated OSNs. *Cophenetic correlation coefficient. See online methods for details of dendrogram generation. [7]: 2-

acetonaphthone, [10]: 2-naphthoïc acid, [11]: benzo[b]thiophene-2-carboxylic acid, [12]: benzo[b]furan-2-carboxylic acid.
